# Relation between Depression Dimensions and Speech Acoustic and Emotion‐based Features

**DOI:** 10.1002/alz70862_110207

**Published:** 2025-12-23

**Authors:** Carolyn W. Zhu, Nadia Ramsingh, Sunghye Cho, Arash Kia, Naomi Nevler, Truda Silberstein, Faye Sheppard, Judith A. Neugroschl, Jimmy Akrivos, Maria Loizos, Margaret Sewell, Carmen Gonzalez‐Recober, Yoon Duk Kim, Daria Birju, Michal S Beeri, Cheryl M Corcoran, Mark Y Liberman, Mary Sano, Laili Soleimani

**Affiliations:** ^1^ Icahn School of Medicine at Mount Sinai, New York, NY USA; ^2^ Linguistic Data Consortium, University of Pennsylvania, Philadelphia, PA USA; ^3^ Perelman School of Medicine, University of Pennsylvania, Philadelphia, PA USA; ^4^ Penn FTD Center, University of Pennsylvania, Philadelphia, PA USA; ^5^ Herbert and Jacqueline Krieger Klein Alzheimer’s Research Center at Rutgers Brain Health Institute, New Brunswick, NJ USA; ^6^ James J. Peters VA Medical Center, Bronx, NY USA

## Abstract

**Background:**

Depression is a common and highly heterogeneous disorder in older adults, often linked to faster cognitive decline. While standard questionnaires are subjective, speech analysis may offer a more objective method for characterizing depression. This study investigates the relationship between speech features and depression dimensions in participants at Mount Sinai Alzheimer’s Disease Research Center (ADRC).

**Method:**

Participants included healthy controls (*n* = 31) and individuals with mild cognitive impairment (MCI= 22) and Alzheimer’s Disease (AD, *n* = 16). They described three pictures with neutral, negative and positive themes. Speech features were analyzed using automated pipelines and emotion‐based variables and acoustic features were used for analysis. Depression dimensions (dysphoria, apathy, hopelessness, and memory complaints) were assessed based on Geriatric Depression Scale‐15 (GDS‐15). Mixed model regression was used to assess the relationship of depression dimensions and the emotional nature of the pictures.

**Result:**

The 73 participants (41% male, average age 80.14±8.01) showed that dysphoria and apathy had opposite associations with emotion‐based measures. Dysphoria was associated with higher valence (more positive emotion), while apathy to lower valence. Subjective memory complaint was also associated with lower valence words.

Further analysis revealed that apathy was associated with lower pitch and slower speech when describing negative pictures, and dysphoria with a wider pitch range and faster speech for negative pictures. Patients with memory complaints used a narrower pitch range in both positive and negative tasks.

**Conclusion:**

Dysphoria was associated with heightened emotional reactivity, while apathy showed decreased reactivity. Apathy and memory complaints shared similar speech features. Our preliminary results support the use of speech features in distinguishing different depression dimensions even at the subclinical level, offering promising opportunities for use of technology to enhance both understanding and diagnosis of depression.

